# Low Incidence of Postoperative Respiratory Depression with Oliceridine Compared to Morphine: A Retrospective Chart Analysis

**DOI:** 10.1155/2020/7492865

**Published:** 2020-10-24

**Authors:** Sergio Bergese, Richard Berkowitz, Paul Rider, Martin Ladouceur, Suzanne Griffith, Alvaro Segura Vasi, Kristina Cochrane, Linda Wase, Mark A. Demitrack, Ashraf S. Habib

**Affiliations:** ^1^Stony Brook University, Stony Brook, NY, USA; ^2^Phoenix Clinical Research, Tamarac, FL, USA; ^3^University of South Alabama Medical Center, Mobile, AL, USA; ^4^Evidera, St-Laurent, QC, Canada; ^5^Research Partners, Inc., Jackson, MS, USA; ^6^North Alabama Medical Center, Florence, AL, USA; ^7^Trevena Inc., Chesterbrook, PA, USA; ^8^Duke University Medical Center, Durham, NC, USA

## Abstract

**Background:**

Oliceridine, an investigational IV opioid, is a first-in-class G-protein selective agonist at the *μ*-opioid receptor. The G-protein selectivity results in potent analgesia with less recruitment of *β*-arrestin, a signaling pathway associated with opioid-related adverse events (ORAEs). In randomized controlled studies in both hard and soft tissue models yielding surgical pain, oliceridine provided effective analgesia with a potential for an improved safety and tolerability profile at equianalgesic doses to morphine. The phase 3, open-label, single-arm, multicenter ATHENA trial demonstrated the safety, tolerability, and effectiveness of oliceridine in moderate to severe acute pain in a broad range of patients undergoing surgery or with painful medical conditions warranting use of an IV opioid. This retrospective, observational chart review study compared respiratory depression events associated with oliceridine administration as found in the ATHENA trial to a control cohort treated with conventional opioids.

**Methods:**

Patients at 18 years of age or older, who underwent colorectal, orthopedic, cardiothoracic, bariatric, or general surgeries between June 2015 and May 2017 in 11 sites participating in the ATHENA trial who received postoperative analgesia either with IV oliceridine or with IV conventional opioids (e.g., morphine alone or in combination with other opioids) (CO cohort); and had a hospital stay >48 hours, were included in this retrospective analysis. Data from the ATHENA trial was used for the oliceridine cohort; and additional baseline characteristics were collected from medical charts. Data from medical charts were collected for all CO cohort patients. The two cohorts were balanced using an inverse probability weighting method. The primary outcome was the incidence of operationally defined opioid-induced respiratory depression (OIRD) in the two cohorts. Secondary outcomes included between-group comparison of the incidence of OIRD events among a subset of high-risk patients.

**Results:**

OIRD was significantly less in the oliceridine cohort compared to the CO cohort (8.0% vs. 30.7%; odds ratio: 0.139) (95% confidence interval [CI] 0.09–0.22; *P* < 0.0001). Likewise, the incidence of OIRD was lower among high-risk patients in the oliceridine cohort (9.1% vs. 34.7%; odds ratio: 0.136) (95% CI [0.09–0.22]; *P* < 0.0001) compared to the CO cohort.

**Conclusion:**

In this retrospective chart review study, patients receiving IV oliceridine for moderate to severe acute pain demonstrated a lower incidence of treatment emergent OIRD compared to patients who were treated with IV morphine either alone or with concomitant administration of other opioids.

## 1. Introduction

In the management of postoperative acute pain, IV opioids remain an important pharmacotherapy, particularly for moderate to severe pain [1–3]. However, conventional opioids are associated with adverse events ranging from pruritus, nausea/vomiting, and constipation to potentially fatal adverse events such as respiratory depression requiring mechanical ventilation [1, 4].

The introduction of multimodal pain management protocols has reduced, though not eliminated, the need for IV opioids [5, 6]. At the same time, drug development efforts have focused on innovative alternatives that may provide opioid-level analgesic efficacy with reduced incidence of opioid-related adverse events (ORAEs). While conventional opioids bind to the *μ*-opioid receptor (MOR) and subsequently engage in postreceptor signaling through G-protein activation, leading to analgesia, it is the concurrent activation of the postreceptor signaling protein *β*-arrestin-2 that has been implicated in the development of ORAEs [7–9]. Thus, development of compounds conferring functional selectivity (biased agonism) with preferential activity in the G-protein signaling pathway over *β*-arrestin pathway has been hypothesized to broaden the therapeutic window, allowing for analgesic effects with fewer adverse events [10, 11].

Oliceridine (Trevena Inc., Chesterbrook, PA, USA) is a next generation G-protein selective agonist at the *μ*-opioid receptor (MOR) that exhibits markedly reduced *β*-arrestin recruitment when compared with conventional opioids [9, 12]. Nonclinical studies and initial clinical trials reported effective, rapid analgesia, with a low incidence of adverse events, with data suggesting less reduction in respiratory drive with oliceridine than morphine [12–16]. In randomized, controlled pivotal phase 3 trials involving hard tissue and soft tissue models of surgical pain, oliceridine IV exhibited a lower incidence of respiratory safety events and gastrointestinal adverse events compared to morphine at doses achieving comparable pain relief [17, 18].

A phase 3, open-label, single-arm, multicenter trial (ATHENA) was conducted in a broad range of patients undergoing a variety of surgeries or receiving treatment for painful medical conditions. The ATHENA trial design used intentionally less restrictive eligibility criteria and treatment protocol requirements. This was done in order to examine the safety and tolerability of the use of oliceridine under circumstances that resembled, as closely as possible, the standard of care in clinical practice in settings where a conventional IV opioid would be indicated for the treatment of moderate to severe acute pain [19].

Findings from the ATHENA trial demonstrated that oliceridine, administered alone or as a component of multimodal analgesia, was generally safe and well tolerated in both surgical and medical patients experiencing moderate to severe acute pain, with nausea (18%), vomiting (7%), and constipation (6%) being the most frequent AEs “probably” or “possibly” related to oliceridine [19], similar to the profile observed in the prior phase 3 controlled trials [17, 18].

The ATHENA trial was a single cohort study and did not include a concurrent control group. Therefore, this retrospective chart review was designed to compare the safety of oliceridine as observed in the ATHENA trial to a control cohort treated with conventional opioids at a subset of the ATHENA investigational sites during the same time period. The objective of this study was to compare safety data, in particular, respiratory depression events between the two cohorts.

## 2. Methods

### 2.1. Ethical Approval and Consent for Participation

The ATHENA trial was approved by the Institutional Review Board (IRB) or Independent Ethics Committee at each investigational site and was conducted in compliance with the ethical principles of the Declaration of Helsinki and all International Conference on Harmonization Good Clinical Practice Guidelines. All patients provided written informed consent before participating in the study.

This retrospective study was approved by the same IRB within each site. The research was conducted in accordance with the approved protocol and complied with the standards of the Declaration of Helsinki. This study involved a retrospective review of the existing data that were deidentified prior to the analysis and was granted a waiver of informed consent by the IRB for the subjects included in the CO cohort.

### 2.2. Overall Study Design

**Figure 1:**
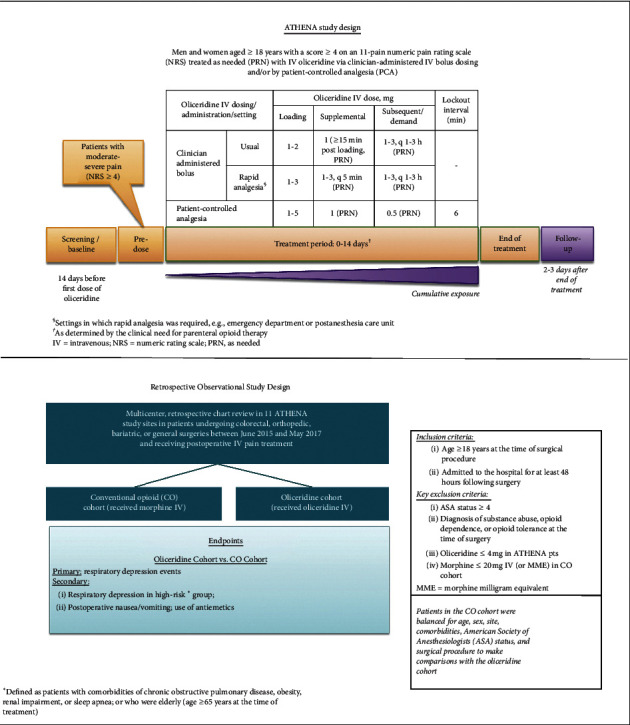
Study design for ATHENA and retrospective observational chart review study. Eleven study sites from the ATHENA trial participated in the retrospective observational chart review study. Subjects in the CO cohort were identified by rigorous chart review during the same interval of time that recruitment for the ATHENA trial had occurred. The CO cohort was limited to individuals treated with IV morphine either alone or in combination with other conventional opioids.

This multicenter, retrospective chart review study was conducted in a subset of the ATHENA investigational sites (11 sites) (Supplemental Table 1). Patients who underwent colorectal, orthopedic, cardiothoracic, bariatric, or general surgeries between June 2015 and May 2017 were included in the analysis. All subjects received analgesia for postoperative pain that included either IV oliceridine (in the original ATHENA trial patients, referred to here as the oliceridine cohort) or IV morphine alone or in combination with other opioids (in the chart review patients, referred to here as the conventional opioid [CO] cohort). The CO cohort was identified by a directed chart review of patients admitted during the same interval of time that ATHENA recruitment had occurred (Figure 1). Site selection was based upon a sufficient size of patient enrollment in the original ATHENA trial at the particular site and the availability of investigational staff at the site to perform the chart review procedures. The safety of oliceridine, with respect to respiratory or gastrointestinal opioid-related adverse events (ORAEs) from the oliceridine cohort, was compared to the same clinical events seen in the CO cohort. In order to enhance the quality of the comparative analysis and allow characterization of the risk profile of the patient populations, the chart review procedure obtained additional clinical details on both cohorts where available in the record, including details of the operative or medical procedures, recovery milestones, and other concomitant treatment interventions. Due to lack of protocol-directed standardized use of antiemetics, the information available on the use of antiemetics from medical records did not provide meaningful information. Thus, only the data obtained for respiratory safety is discussed in this manuscript.

### 2.3. Oliceridine Cohort Patient Population

The ATHENA trial enrolled patients at 18 years of age or older, experiencing moderate to severe acute pain following surgery or accompanying a painful nonsurgical medical condition, defined as a score ≥4 on an 11-point numeric rating scale (NRS) [ranging from 0 = no pain to 10 = worst pain]. Patients with an American Society of Anesthesiologists (ASA) physical status of ≥4 at the time of surgery were excluded. In addition, patients with a diagnosis of substance abuse, opioid dependence, or opioid tolerance at the time of surgery were excluded. The trial enrolled a total of 768 patients with mean age 54.1 ± 16.1 years and a mean pain score of 6.3 ± 2.1 (NRS) who received oliceridine IV. One-third of the enrolled patients (32.2%) were ≥65 years old and half of all patients (45.8%) had a body mass index (BMI) ≥ 30 kg/m^2^. Most patients were female (64.8%) and Caucasian (77.8%). Surgical patients comprised the majority of the study population (93.9%), most common being orthopedic (30.1%), colorectal (15.0%), or gynecologic (15.0%) procedures. A detailed presentation of the study design and results for the ATHENA trial are reported elsewhere [19].

Eligible patients from 11 sites participating in the ATHENA trial comprised the oliceridine cohort used in this retrospective chart review study. To meet the inclusion criteria for this analysis, these ATHENA patients were of age 18 years or older at the time of surgical procedure, with ASA physical status less than 4 and admitted in the hospital for at least 48 hours following surgery. They should have received one or more doses of IV oliceridine (minimum cumulative dose of 4 mg) for the management of postoperative pain during the inpatient hospital stay, including time spent in the postanesthesia care unit (PACU) (Figure 1).

### 2.4. Oliceridine Treatment Protocol

Oliceridine cohort patients were treated with intravenous (IV) oliceridine via clinician-administered bolus dosing and/or patient-controlled analgesia (PCA) (Figure 1). For IV bolus dosing, a loading dose of 1 to 2 mg was administered, and a supplemental dose of 1 mg was given within 15 minutes if needed. In settings where rapid analgesia was required (e.g., emergency departments, PACU), loading doses of 1 to 3 mg were administered and supplemental doses of 1 to 3 mg every 5 minutes PRN were allowed. Subsequent doses of 1 mg to 3 mg every 1 to 3 hours were administered as needed (PRN). For PCA, a loading dose of 1.5 mg and demand dose of 0.5 mg were administered using a 6-minute lockout interval. If clinically indicated, 1 mg supplemental doses were allowed PRN throughout the remainder of the treatment period. Treatment duration for each patient was determined by the clinical need for parenteral opioid therapy.

Patients could have received IV oliceridine as a part of a multimodal analgesia protocol in place at the investigational site, which may have included the use of NSAIDs, glucocorticoids, gabapentin, or pregabalin. Per protocol, concomitant use of other conventional opioids along with oliceridine IV was not permitted; however, patients could receive other IV or oral opioids after the administration of the last dose of oliceridine. Opioids received after the last dose of oliceridine included hydromorphone, hydrocodone (with or without acetaminophen), and oxycodone (with or without acetaminophen).

### 2.5. CO Cohort Patient Population

Patients eligible for inclusion in the CO cohort were of age 18 years or older at the time of surgical procedure, with an ASA physical status less than 4 and had a duration of admission to the hospital for at least 48 hours following their surgery. Eligible patients also should have received one or more doses of IV morphine (minimum dose of 20 mg morphine equivalent [MME] per day) for the management of postoperative pain during the inpatient hospital stay, including time spent in the PACU. Finally, patients having a diagnosis of substance abuse, opioid dependence, or opioid tolerance at the time of surgery were excluded.

Using the inverse probability weighting (IPW) approach (discussed below), patients in the CO cohort were balanced with those in the oliceridine cohort in several prespecified characteristics in the multivariable analyses, including age, sex, site, illness comorbidities, ASA physical status, and type of surgical procedure. To further assure comparability, the timeframe for index events was required to overlap with the enrollment period for ATHENA trial.

### 2.6. Treatment in the CO Cohort Patient Population

All patients in the CO cohort could have received IV morphine as a part of a multimodal analgesia protocol in existence at their study site. Patients could have received one or more conventional opioids anytime during the postoperative period. This additional opioid may or may not have been administered concurrently with the primary opioid, IV morphine. Other conventional opioids received included hydromorphone (IV and oral), meperidine, hydrocodone/acetaminophen, oxycodone, and tramadol.

### 2.7. Inverse Probability Weighting (IPW) Methodology

Inverse probability weighting (IPW) was used to address potential imbalances among covariates between the two cohorts. The IPW approach weights patient observations based on a propensity score, which is defined as the weighted probability of a patient belonging to a specific cohort. Propensity scores were estimated using a multivariable logistic regression model conditional on the observed baseline covariates (surgical procedure type, ASA status, age, sex, race [characterized as white/nonwhite], and body mass index [BMI]).

By weighting patients using the inverse of propensity scores (i.e., the probability of treatment), the cohorts then have improved balance on observable covariates across treatment groups.

In a subset of the comparative samples (*N* = 95 pairs), a direct one-to-one (1 : 1) matching approach was possible using the same observed matching variables as those in the complete IPW balanced sample. A sensitivity analysis was performed in this subset of 95 direct-matched pairs of patients as a confirmatory analysis of the findings in the overall IPW population analysis.

### 2.8. Safety Outcomes

#### 2.8.1. Primary Outcome

The primary outcome was the incidence of operationally defined opioid-induced respiratory depression (OIRD). Because the method of ascertainment of OIRD differed between the two cohorts, a prespecified, comprehensive operational definition of OIRD was applied to each cohort in order to create a comparable set of terms for this event in each group.

For the oliceridine cohort, OIRD events were prespecified, as defined in the original protocol, for all patients based on verbatim clinical terms coded using adverse event methodology defined in MedDRA reporting terminology (MedDRA, version 19.0). MedDRA coded terms included terms for hypoventilation, hypoxia, respiratory depression, and respiratory failure categories and contained over 100 possible MedDRA-coded terms in the analysis.

For the CO cohort, ICD-9 and ICD-10 codes were obtained as recorded in each patient's chart and entered into their accompanying Electronic Medical Record (EMR). At least 29 possible codes were examined, including abnormal, diminished, or shallow breath sounds, oxygen desaturation and/or hypoxia, dyspnea, respiratory failure, respiratory insufficiency, respiratory compromise, and other respiratory terms (Supplemental Table 2).

#### 2.8.2. Secondary Outcomes

The secondary outcomes were (a) a between-group comparison of the incidence of OIRD events among a subset of high-risk patients, defined as patients with a comorbid medical diagnosis of chronic obstructive pulmonary disease, obesity, renal impairment, or sleep apnea, or who were elderly (age ≥ 65 years at the time of treatment).

### 2.9. Statistical Analysis

A sample size target of 250 patients within each cohort was selected based on a projected respiratory depression probability of 14.5% for the CO cohort vs. 7.25% for the oliceridine group. The projected event rates were calculated using data from the phase 2b and phase 3 clinical trials with oliceridine [15, 17, 18]. This sample size calculation also incorporated an 80% power, an alpha of 0.05 (one-sided), and a missing data rate of 10%.

Descriptive statistics of baseline variables are provided. Continuous variables were described by mean, median, interquartile range (IQR), standard deviation (SD), minimum, and maximum. Categorical variables were described by frequency and percentages (*n*, %).

Statistical comparisons between the oliceridine and CO cohorts were made with *t*-tests or chi-square tests. Incomplete/missing data were not imputed, except for partially missing dates. All analyses were performed using SAS v9.4 (SAS Institute Inc., Cary, NC, USA).

## 3. Results

**Figure 2:**
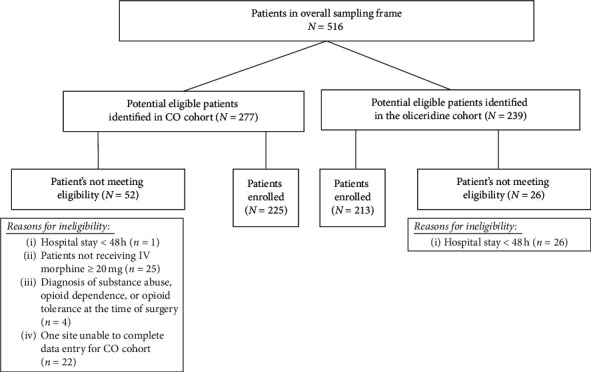
Patient disposition.

A final population of 225 patients in the CO cohort and 213 patients in the oliceridine cohort met the eligibility criteria and were included in the analyses (Figure 2). There were no significant differences in racial distribution, mean BMI, BMI categories of <30 kg/m^2^ and ≥30 kg/m^2^, or proportion of patients considered as high-risk between the 2 cohorts (Table 1). Patients were of mean age 62.6 ± 12.3 years in the CO cohort and 60.1 ± 13.8 years in oliceridine cohort, *P* = 0.04. The proportion of elderly patients (age ≥ 65 years) was higher in the oliceridine cohort (51.6%, *n* = 110, vs. 41.3%, *n* = 93; *P* = 0.04). In the oliceridine cohort, 38% (*n* = 81) had ASA physical status 3, while, in the CO cohort, 71.6% (*n* = 161) of patients had an ASA physical status 3 (*P* < 0.001). The use of opioids in the 12 months prior to the current hospitalization was unknown in 45% of patients in the oliceridine cohort (vs. 4.0% in the CO cohort); however, based on the information available, prior opioid use was significantly higher in the CO cohort vs. oliceridine cohort (14.1%, *n* = 30, for oliceridine cohort vs. 35.1%, *n* = 79, in the CO cohort; *P* < 0.0001). There was a significantly higher proportion of patients undergoing colorectal surgery enrolled in the oliceridine cohort (46.0%, *n* = 98, for oliceridine cohort vs. 33.3%, *n* = 75, for CO cohort; *P* = 0.01). Among patients undergoing orthopedic surgery, 36.6% (*n* = 78) were enrolled in the oliceridine cohort vs. 53.3% (*n* = 120) in the CO cohort (*P* = 0.0006). Overall, 80.1% (*n* = 351) of patients were described as meeting the “higher risk” criteria, with obese (50.7%, *n* = 222) and elderly (46.4%, *n* = 203) subpopulations being the most common. Distributions of patients with high risk were similar across the individual cohorts except for elderly (as reported above).

### 3.1. Exposure to Oliceridine and Conventional Opioids

In the ATHENA trial protocol, concomitant use of opioids was not permitted among patients treated with IV oliceridine except after termination of treatment with oliceridine. In contrast, 91.6% (*n* = 206) of patients in the CO cohort received opioids concomitantly or after treatment with IV morphine, with hydromorphone as the most commonly used opioid among 26.7% (*n* = 82) of patients. In the oliceridine cohort, 32.8% (*n* = 170) of patients received oral opioids after the last dose of oliceridine. The mean (SD) cumulative dose of IV oliceridine was 51.4 (38.1) mg [median dose: 42.5 mg] in the oliceridine cohort and that of IV morphine with other conventional opioids was 112.1 (99.4) mg [median dose: 79.2 mg] in the CO cohort (Table 2).

### 3.2. Primary Outcome

**Figure 3:**
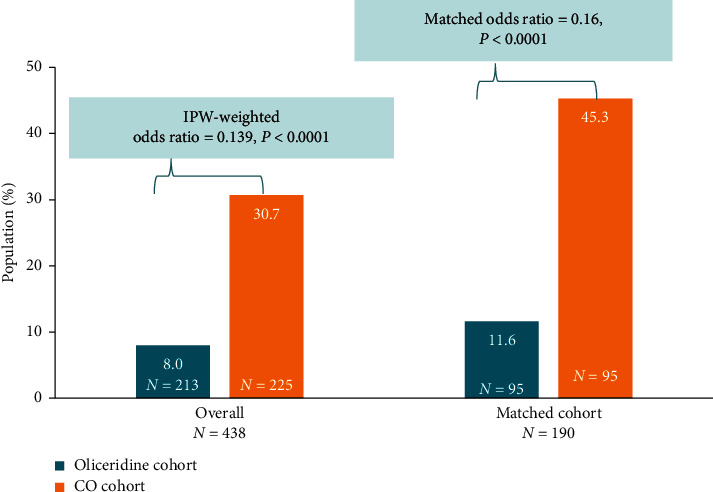
Incidence of opioid-induced respiratory depression (primary outcome). Using the Inverse probability weighting (IPW) approach patients in the CO cohort were balanced with those in the oliceridine cohort in several prespecified characteristics, including age, sex, site, illness comorbidities, American Society of Anesthesiologists (ASA) status, and type of surgical procedure. The IPW approach weights patient observations based on a propensity score, which is defined as the weighted probability of a patient belonging to a specific cohort. Propensity scores were estimated using a multivariate logistic regression model conditional on the observed baseline covariates (surgical procedure type, ASA status, age, sex, race [characterized as white/nonwhite], and BMI). Matched cohort consisted of 95 subjects in either cohort with a direct one-to-one (1 : 1) matching approach on the same observed matching variables used in the complete IPW balanced sample. For the oliceridine cohort, OIRD events were prespecified, as defined in the original study protocol for all patients based on verbatim clinical terms coded using adverse event methodology defined in MedDRA reporting terminology (MedDRA, version 19.0). For the CO cohort, ICD-9 and ICD-10 codes were obtained as recorded in each patient's chart review and entered into their accompanying Electronic Medical Record (EMR). A total of 206 patients (91.6%) in the CO cohort received other opioids concomitantly or after treatment with IV morphine. Incidence of respiratory events was significantly lower in the oliceridine cohort.

With the IPW method adjusting the imbalances on the covariates at baseline, patients treated with IV oliceridine had 86% lower odds (odds ratio [OR] = 0.139, 95% confidence interval [CI] [0.09–0.22], *P* < 0.0001) of experiencing a respiratory depression event during the postoperative hospitalization. Similar results were observed in the direct-matched cohort sensitivity analysis (OR 0.160; 95% CI [0.08–0.34], *P* < 0.0001) (Figure 3).

### 3.3. Secondary Outcomes

**Figure 4:**
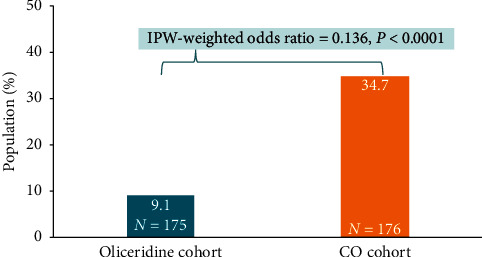
Incidence of opioid-induced respiratory depression in high-risk subgroup (secondary outcome). High-risk patients were defined as patients who were obese, or with a comorbid medical diagnosis of chronic obstructive pulmonary disease, renal impairment, or sleep apnea, or who were elderly (age ≥ 65 years at the time of treatment). Incidence of respiratory events was significantly lower in the oliceridine cohort in the high-risk subgroup.

Among the subset of high-risk patients (with any of the following: COPD, obesity, renal impairment, sleep apnea, or elderly), the incidence of OIRD was lower among patients in the oliceridine cohort compared to patients treated with conventional opioids in the CO cohort (9.1% vs. 34.7%), with an adjusted OR of 0.136 (95% CI [0.09–0.22]; *P* < 0.0001) (Figure 4).

## 4. Discussion

This retrospective case-control study evaluated the safety outcomes of oliceridine compared with conventional opioids (IV morphine alone or in combination with other opioids in 92% of the patients) on key opioid-induced adverse events. Several steps were taken to select the comparison population for this analysis. First, we limited the comparison population to individuals who were treated with IV morphine either alone or in combination with other opioids (excluding the use of oliceridine) for similar clinical indications at a subset of the sites participating in the ATHENA trial. Second, subjects were identified by chart review during the same interval of time that ATHENA recruitment had occurred. Finally, we used an inverse probability weighting propensity score methodology to achieve an appropriate balance of the two study cohorts for the analysis of outcomes.

Findings from the IPW method show that patients treated with IV oliceridine were less likely to experience OIRD compared to CO cohort (IV morphine alone or in combination with other opioids) (8.0% vs. 30.7% in the CO cohort, OR 0.139, *P* < 0.0001). Although the baseline characteristics differed in the two cohorts, the results were consistent when using a direct-matching approach, where the 2 cohorts were matched 1 : 1 for baseline variables—surgical procedure, ASA physical status, age, sex, race, and BMI. In addition, the findings were also observed in a subgroup of patients considered high-risk (patients who were obese or elderly, or with medical comorbidities of chronic pulmonary disease or renal impairment or sleep apnea).

Based on early clinical findings, the approximate dose equivalency of oliceridine versus morphine after the initial dose is 1 mg oliceridine: 5 mg morphine (data on file). However, the dose equivalency of oliceridine to morphine after cumulative dosing has not been established. In this study, the median cumulative dose was 43 mg IV oliceridine in the oliceridine cohort and 79 mg IV morphine equivalents in the CO cohort. In addition, in patients experiencing an event of respiratory depression, the median cumulative dose of IV oliceridine was 35 mg and the dose of IV morphine with concomitant opioids was 74 MME in the CO cohort. Previous meta-analyses on the risk factors for OIRD in surgical patients have reported morphine equivalent daily dose of 24.7 ± 14 (mean ± SD) mg in patients experiencing OIRD [20]. A prior investigation found that total opioid dose at 72 hours predicts risk of postoperative sleep-disordered breathing and the opioid median cumulative dose reported at 72 hours was 55 mg IV morphine-equivalent dose [21]. In our study, the median cumulative dose of IV conventional opioids in patients experiencing OIRD was 74 MME. It was difficult to obtain the cumulative dose by day, as the data available in the medical record was usually recorded as “start date” of treatment and “end date” of treatment. Nevertheless, the dose of conventional opioids in our study associated with a respiratory event was higher than those reported in the literature. However, it is important to note that opioid dose alone does not determine the risk. Indeed, during IV PCA use, rapid-dose escalations and patient-specific variables, such as older age and type of surgery, can also influence the risk of OIRD [20, 22].

The findings on the lower rates of OIRD in the oliceridine cohort compared to CO cohort using IV morphine alone or combined with conventional opioids are consistent with those observed in randomized clinical phase 3 trials of patients following a bunionectomy or abdominoplasty procedure, where a reduced rate of respiratory events was reported with IV oliceridine compared with morphine [17, 18].

In this retrospective analysis, respiratory depression using MedDRA events was recorded in the oliceridine cohort while ICD-9/ICD-10 codes were used to report the events of respiratory depression in the CO cohort. The rates of respiratory depression events seen with IV morphine alone or combined with conventional opioids (30.7% in the overall patients; 45.3% in the direct-matched group) in our study are similar to those reported by Shafi et al. [1] and the PRODIGY trial [23]. In their retrospective analysis, conducted from a large healthcare system including 21 acute care hospitals and 135,379 patients undergoing surgical and endoscopic procedures and receiving opioids, Shafi et al. used ICD-9 codes to define ORAEs, including respiratory events, and reported an incidence as high as 49% [1]. Likewise, in the PRODIGY trial, an international, prospective observational study, respiratory depression events [defined as etCO_2_ ≤ 15 or ≥ 60 mm Hg for ≥ 3 mins, RR ≤ 5 breaths for ≥ 3 mins, SpO_2_ ≤ 85% for ≥ 3 mins, apnea > 30 sec] were reported in 46% of patients [23]. In the current study, the rates of OIRD reported for the oliceridine cohort (8.0% in the overall group and 11.6% in the matched group) are similar to the incidence of respiratory safety events reported in phase 3 randomized clinical trials with oliceridine [17, 18].

In our study, the high-risk subgroup included patients who were obese or elderly, or with medical comorbidities of chronic pulmonary disease, renal impairment, or sleep apnea. All these factors are considered as significant risk factors for postoperative OIRD [24, 25]. In patients with these underlying risk factors, it is recommended to carefully titrate the dose of opioids, in addition to careful monitoring [24]. Our findings that the high-risk subgroup patients are less likely to experience OIRD with oliceridine compared to IV morphine alone or combined with conventional opioids are encouraging.

### 4.1. Limitations

There are several notable limitations to this report, including the retrospective design as well as reliance on the healthcare professional (physician/nurse) documentation of all data in the Electronic Medical Records (EMR). The EMR did not document precisely as to whether other opioids were administered concomitantly or sequentially leading to a potential bias on estimating the risk of OIRD resulting from the concomitant use of opioids in the CO cohort. Likewise, due to incomplete documentation, the influence of anesthetics used during surgery on the incidence of respiratory depression could not be evaluated. An important limitation was the inability to standardize the operational definition of OIRD between the two cohorts which may have resulted in a difference in case ascertainment between the groups. There was also inadequate information on the use of opioids in the previous year prior to hospitalization, including acute or chronic use, particularly for patients in the oliceridine cohort. This is a limitation as chronic use of opioids can further confound the results [26].

## 5. Conclusions

In this retrospective chart review study, patients receiving oliceridine for the management of their acute moderate to severe pain were observed to experience a lower incidence of treatment associated OIRD compared to a population of patients who had received IV morphine either alone or with concomitant administration of other opioids. These findings were also observed among a high-risk population of patients who were elderly and had significant medical comorbidities complicating their clinical condition.

Overall, the findings are consistent with the underlying pharmacologic profile of oliceridine and suggest that this compound may provide a potentially important new option for patients with postoperative pain where the use of an IV opioid medication is warranted. Additional prospective studies are needed to more fully evaluate the potential improvements in respiratory safety observed here.

## Figures and Tables

**Table 1:** Demographic and baseline characteristics.

Characteristic	Oliceridine cohort (*n* = 213)	CO cohort (*n* = 225)	*P* value^*∗∗*^
Female gender	125 (58.7)	112 (49.8)	0.076

Age, years, mean (SD)	62.6 (12.3)	60.1 (13.8)	0.039
<65 years	103 (48.4)	132 (58.7)	0.039
≥65 years	110 (51.6)	93 (41.3)	0.039

Race			
White	159 (74.7)	181 (80.4)	0.180
Black/African American	47 (22.1)	37 (16.4)	0.170
Other	4 (1.9)	2 (0.9)	0.632
Unknown	3 (1.4)	5 (2.2)	

ASA status			
1	11 (5.2)	7 (3.1)	0.400
2	121 (56.8)	57 (25.3)	<0.0001
3	81 (38.0)	161 (71.6)	<0.0001

BMI^*∗*^, kg/m^2^, mean (SD)	31.2 (7.9)	31.5 (7.8)	0.689
<30 kg/m^2^	106 (49.8)	107 (47.6)	0.817
≥30 kg/m^2^	107 (50.2)	115 (51.1)	0.817

Surgery type			
Bariatric	2 (0.9)	0 (0)	0.455
Colorectal	98 (46.0)	75 (33.3)	0.009
General	35 (16.4)	30 (13.3)	0.437
Orthopedic	78 (36.6)	120 (53.3)	0.0006

CORES (%)	175 (82.2)	176 (78.2)	0.362
COPD	38 (17.8)	42 (18.7)	0.920
Obesity	107 (50.2)	115 (51.1)	0.930
Renal impairment	10 (4.7)	5 (2.2)	0.246
Elderly	110 (51.6)	93 (41.3)	0.039
Sleep apnea	41 (19.3)	36 (16.0)	0.443

Opioid use 12 months prior to hospitalization	30 (14.1)	79 (35.1)	<0.0001
Information unknown	96 (45.1)	9 (4.0)	

^*∗*^3 patients had missing BMI and excluded from the model; COPD = chronic obstructive pulmonary disease. Data are presented as *n* (%), except where specified. ^*∗∗*^*P* values were computed from *t*-test for age and BMI and from chi-square test for gender, age categories, race, ASA status, BMI categories, surgical type, and CORES status.

**Table 2:** Cumulative dose of oliceridine and conventional opioids.

Cumulative dosing		Oliceridine cohort	CO cohort^*∗*^
All patients	*N*	213	225
	Dose, mg, mean (SD)	51.4 (38.1)	112.1 (99.4)
	Dose, mg, median	42.5	79.2

Patients experiencing respiratory depression	*n* (%)	17 (8.0)	69 (30.7)
	Dose, mg, mean (SD)	40.7 (26.6)	96.5 (69.0)
	Dose, mg, median	35	74

^*∗∗*^Cumulative dosage for morphine + hydromorphone + hydrocodone + meperidine + oxycodone + tramadol in MME (patients may have one or more of these treatments).

## Data Availability

The data used to support the findings of this study are available from the sponsor, Trevena, Inc.., upon request.

## Supplementary Materials

Supplementary Materials Supplemental Table 1: list of participating sites and number of patients enrolled. Supplemental Table 2: MedDRA codes for respiratory depression used in oliceridine cohort and ICD-9/ICD-10 codes for respiratory depression used for the SOC cohort.

## References

[1] Shafi S., Collinsworth A. W., Copeland L. A. (2018). Association of opioid-related adverse drug events with clinical and cost outcomes among surgical patients in a large integrated Health care delivery system. *JAMA Surgery*.

[2] Gan T. J., Epstein R. S., Leone-Perkins M. L., Salimi T., Iqbal S. U., Whang P. G. (2018). Practice patterns and treatment challenges in acute postoperative pain management: a survey of practicing physicians. *Pain and Therapy*.

[3] Colvin L. A., Bull F., Hales T. G. (2019). Perioperative opioid analgesia-when is enough too much? A review of opioid-induced tolerance and hyperalgesia. *The Lancet*.

[4] Mallappallil M., Sabu J., Friedman E., Salifu M. (2017). What do we know about opioids and the kidney?. *International Journal of Molecular Sciences*.

[5] Chou R., Gordon D. B., de Leon-Casasola O. A. (2016). Management of postoperative pain: a clinical practice guideline from the American pain society, the American society of regional anesthesia and pain medicine, and the American society of Anesthesiologists’ committee on regional anes’hesia, executive committee, and administrative council. *The Journal of Pain*.

[6] Wardhan R., Chelly J. (2017). Recent advances in acute pain management: understanding the mechanisms of acute pain, the prescription of opioids, and the role of multimodal pain therapy. *F1000Research*.

[7] Bohn L. M., Lefkowitz R. J., Gainetdinov R. R., Peppel K., Caron M. G., Lin F.-T. (1999). Enhanced morphine analgesia in mice lacking -arrestin 2. *Science*.

[8] Raehal K. M., Walker J. K. L., Bohn L. M. (2005). Morphine side effects in *β*-arrestin 2 knockout mice. *Journal of Pharmacology and Experimental Therapeutics*.

[9] DeWire S. M., Yamashita D. S., Rominger D. H. (2013). A G protein-biased ligand at the *μ*-opioid receptor is potently analgesic with reduced gastrointestinal and respiratory dysfunction compared with morphine. *Journal of Pharmacology and Experimental Therapeutics*.

[10] Raehal K. M., Schmid C. L., Groer C. E., Bohn L. M. (2011). Functional selectivity at the *μ*-opioid receptor: implications for understanding opioid analgesia and tolerance. *Pharmacological Reviews*.

[11] Schmid C. L., Kennedy N. M., Ross N. C. (2017). Bias factor and therapeutic window correlate to predict safer opioid analgesics. *Cell*.

[12] Soergel D. G., Subach R. A., Burnham N. (2014). Biased agonism of the *μ*-opioid receptor by TRV130 increases analgesia and reduces on-target adverse effects versus morphine: a randomized, double-blind, placebo-controlled, crossover study in healthy volunteers. *Pain*.

[13] Soergel D. G., Ann Subach R., Sadler B. (2014). First clinical experience with TRV130: pharmacokinetics and pharmacodynamics in healthy volunteers. *The Journal of Clinical Pharmacology*.

[14] Violin J. D., Crombie A. L., Soergel D. G., Lark M. W. (2014). Biased ligands at G-protein-coupled receptors: promise and progress. *Trends in Pharmacological Sciences*.

[15] Singla N., Minkowitz H., Soergel D. (2017). A randomized, Phase IIb study investigating oliceridine (TRV130), a novel *μ*-receptor G-protein pathway selective (*µ*-GPS) modulator, for the management of moderate to severe acute pain following abdominoplasty. *Journal of Pain Research*.

[16] Viscusi E. R., Webster L., Kuss M. (2016). A randomized, phase 2 study investigating TRV130, a biased ligand of the *μ*-opioid receptor, for the intravenous treatment of acute pain. *Pain*.

[17] Singla N. K., Skobieranda F., Soergel D. G. (2019). APOLLO-2: a randomized, placebo and active-controlled phase III study investigating oliceridine (TRV 130), a G protein-biased ligand at the *μ*-opioid receptor, for management of moderate to severe acute pain following abdominoplasty. *Pain Practice*.

[18] Viscusi E. R., Skobieranda F., Soergel D. G., Cook E., Burt D. A., Singla N. (2019). APOLLO-1: a randomized placebo and active-controlled phase III study investigating oliceridine (TRV130), a G protein-biased ligand at the micro-opioid receptor, for management of moderate-to-severe acute pain following bunionectomy. *Journal of Pain Research*.

[19] Bergese S. D., Brzezinski M., Hammer G. B. (2019). ATHENA: a phase 3, open-label study of the safety and effectiveness of oliceridine (TRV130), A G-protein selective agonist at the *μ*-opioid receptor, in patients with moderate to severe acute pain requiring parenteral opioid therapy. *Journal of Pain Research*.

[20] Gupta K., Nagappa M., Prasad A. (2018). Risk factors for opioid-induced respiratory depression in surgical patients: a systematic review and meta-analyses. *BMJ Open*.

[21] Chung F., Liao P., Elsaid H., Shapiro C. M., Kang W. (2014). Factors associated with postoperative exacerbation of sleep-disordered breathing. *Anesthesiology*.

[22] Jarzyna D., Jungquist C. R., Pasero C. (2011). American Society for Pain Management Nursing guidelines on monitoring for opioid-induced sedation and respiratory depression. *Pain Management Nursing*.

[23] Khanna A. K., Bergese S. D., Jungquist C. R. (2020). Prediction of opioid-induced respiratory depression on inpatient wards using continuous capnography and oximetry: an international prospective, observational trial. *Anesthesia and Analgesia*.

[24] Gupta K., Prasad A., Nagappa M., Wong J., Abrahamyan L., Chung F. F. (2018). Risk factors for opioid-induced respiratory depression and failure to rescue: a review. *Current Opinion in Anaesthesiology*.

[25] Dahan A., Aarts L., Smith T. W. (2010). Incidence, reversal, and prevention of opioid-induced respiratory depression. *Anesthesiology*.

[26] Jungquist C. R., Smith K., Nicely K. L., Polomano R. C. (2017). Monitoring hospitalized adult patients for opioid-induced sedation and respiratory depression. *American Journal of Nursing*.

