# Supplementary material for: Low Incidence of Postoperative Respiratory Depression with Oliceridine Compared to Morphine: A Retrospective Chart Analysis
Source: Pain Res Manag. 2020 Oct 24;2020:7492865. doi: 10.1155/2020/7492865 (PMC7604609; doi:10.1155/2020/7492865)
Supplement: Supplementary Materials — Supplemental Table 1: list of participating sites and number of patients enrolled. Supplemental Table 2: MedDRA codes for respiratory depression used in oliceridine cohort and ICD-9/ICD-10 codes for respiratory depression used for the SOC cohort. [file 7492865.f1.docx]

**Title: Low Incidence of Postoperative Respiratory Depression with Oliceridine Compared to Morphine: A Retrospective Chart Analysis**

**Authors:** Sergio Bergese^1^; Richard Berkowitz^2^; Paul Rider^3^; Martin Ladouceur^4^, Suzanne Griffith^5^, Alvaro Segura Vasi^6^, Kristina Cochrane^7^, Linda Wase^7^; Mark A Demitrack^7^; Ashraf S Habib^8^

^1^Stony Brook University; Stony Brook, NY

^2^Phoenix Clinical Research, Tamarac, FL

^3^University of South Alabama Medical Center, Mobile, AL,

^4^Evidera, St-Laurent, QC, Canada

^5^Research Partners, Inc., Jackson, MS

^6^North Alabama Medical Center, Florence, AL

^7^Trevena, Inc., Chesterbrook, PA;

^8^Duke University Medical Center, Durham, NC

**Corresponding author:**

Ashraf S Habib

Department of Anesthesiology,

Duke University,

2301 Erwin Road, Durham, NC, 27710, USA,

Phone: Tel +1 919 668 2024,

Fax: Fax +1 919 681 4698

Email:[ashraf.habib@duke.edu](mailto:ashraf.habib@duke.edu)

Key words: postoperative, acute pain, analgesia, patient-controlled, opioid-induced respiratory depression

**Supplemental Table 1: List of Participating Sites and Number of Patients Enrolled**

| **Site** | **Number enrolled** |
| --- | --- |
| Horizon Research Partners, LLC 3610 Springhill Memorial Drive North Mobile, AL 36608 | 123 |
| MS Baptist Medical Center 1225 North State Street Jackson, MS 39202 | 71 |
| Phoenix Clinical Research 7171 North University Drive Tamarac, FL 33321 | 58 |
| Shoals Clinical Research Associates, LLC 205 Marengo Street Florence, AL 35630 | 39 |
| Pensacola Research Consultants 4700 Bayou Boulevard Pensacola, FL 32503 | 36 |
| Shoals Medical Trials, Inc. 1300 South Montgomery Avenue Sheffield, AL 35660 | 32 |
| The OH State University Wexner Medical Center 410 W 10th Avenue N411 Columbus, OH 43210 | 29 |
| University of South AL Medical Center 2451 Fillingim Street, Suite 101 Mobile, AL 36617 | 24 |
| TX Orthopedic Specialists, PLLC 2425 Highway 121 Bedford, TX 76021 | 12 |
| Christus Santa Rosa Hospital Alamo Heights 1200 Brooklyn Avenue, Suite 150 San Antonio, TX 78209 | 9 |
| Rush University Medical Center 1653 W. Congress Parkway Chicago, IL 60612 | 5 |

Supplement Table 2: MedDRA codes for respiratory depression used in Oliceridine Cohort and ICD-9/ICD-10 codes for respiratory depression used for the SOC cohort

| Oliceridine Cohort- MedDRA codes | SOC Cohort -ICD-9/ICD-10 codes |
| --- | --- |
| Acute respiratory distress syndrome  Acute respiratory failure  Apnoea  Apnoeic attack  Bradypnoea  Breath holding  Breath sounds abnormal  Breath sounds absent  Cardio-respiratory distress  Central-alveolar hypoventilation  Chronic respiratory failure  Central-alveolar hypoventilation  Chronic respiratory failure  Hypopnoea  Hypoventilation  Hypoventilation neonatal  Infantile apnoea  Lung hypoinflation  Meconium aspiration syndrome  Neonatal respiratory arrest  Neonatal respiratory depression  Neonatal respiratory distress syndrome  Neonatal respiratory failure  Postoperative respiratory distress  Postoperative respiratory failure  Respiratory arrest  Respiratory depression  Respiratory depth decreased  Respiratory distress  Respiratory failure  Respiratory paralysis  Respiratory rate decreased  Severe acute respiratory syndrome  Alveolar oxygen partial pressure abnormal  Alveolar oxygen partial pressure decreased  Alveolar-arterial oxygen gradient increased  Anoxia | ICD-9 Codes  514 Pulmonary congestion and hypostasis  518.52 Other pulmonary insufficiency, not elsewhere classified, following trauma and surgery  518.53 Acute and chronic respiratory failure following trauma and surgery  518.81 Acute respiratory failure  518.82 Other pulmonary insufficiency, not elsewhere classified  786.09 Other dyspnea and respiratory abnormality  799.02 Hypoxemia  799.1 Respiratory arrest  ICD-10 Codes  J95.1 Acute pulmonary insufficiency following thoracic surgery  J95.2 Acute pulmonary insufficiency following nonthoracic surgery  J95.3 Chronic pulmonary insufficiency following surgery  J95.821 Acute postprocedural respiratory failure  J95.822 Acute and chronic postprocedural respiratory failure  J95.89 Other postprocedural complications and disorders of respiratory system, not elsewhere classified  J96.00 Acute respiratory failure, unspecified whether with hypoxia or hypercapnia  J96.01 Acute respiratory failure with hypoxia  J96.02 Acute respiratory failure with hypercapnia  J96.20 Acute and chronic respiratory failure, unspecified whether with hypoxia or hypercapnia  J96.21 Acute and chronic respiratory failure with hypoxia  J96.22 Acute and chronic respiratory failure with hypercapnia  J96.90 Respiratory failure, unspecified, unspecified whether with hypoxia or hypercapnia  J96.91 Respiratory failure, unspecified with hypoxia  J96.92 Respiratory failure, unspecified with hypercapnia  R0600 dyspnea, unspecified  R063 Other forms of dyspnea  R063 Periodic breathing  R0689 other abnormalities of breathing  R09.02 Hypoxemia  R09.2 Respiratory arrest |
